# Supplementary material for: Nanopore-based targeted next-generation sequencing (tNGS): A versatile technology specialized in detecting low bacterial load clinical specimens
Source: PLoS One. 2025 May 27;20(5):e0324003. doi: 10.1371/journal.pone.0324003 (PMC12111578; doi:10.1371/journal.pone.0324003)
Supplement: Attachment — Submitted filename: Response to Reviewers.pdf [file pone.0324003.s003.pdf]

Editor:

The changes in our revised manuscript are highlighted in blue, and the locations of corrections or supplements are specifically indicated in the corresponding responses. Thank you for checking again.

1. Please ensure that your manuscript meets PLOS ONE's style requirements, including those for file naming.

We have adjusted the style of the article by searching for previous papers published in "PLOS ONE". Please review again.

2. Please state what role the funders took in the study. If the funders had no role, please state: "The funders had no role in study design, data collection and analysis, decision to publish, or preparation of the manuscript.

We have provided additional explanations in online system. Please double check.

3. All PLOS journals now require all data underlying the findings described in their manuscript to be freely available to other researchers, either a. In a public repository, b. Within the manuscript itself, or c. Uploaded as supplementary information.

We have presented all data used in this study in the NGDC Bioproject, accession number PRJCA034394, and corrected the statement of data availability(Page 21). Please double check.

4.PLOS requires an ORCID iD for the corresponding author in Editorial Manager on papers submitted after December 6th, 2016. Please ensure that you have an ORCID iD and that it is validated in Editorial Manager. To do this, go to 'Update my Information' (in the upper left-hand corner of the main menu), and click on the Fetch/Validate link next to the ORCID field. This will take you to the ORCID site and allow you to create a new iD or authenticate a pre-existing iD in Editorial Manager.

ORCID: 0009-0003-9871-238X

5. Your ethics statement should only appear in the Methods section of your

manuscript. If your ethics statement is written in any section besides the Methods, please delete it from any other section.

Thank you for checking again.

Reviewer 1：

1.The methodology section mentions IGRA as one of the diagnostic criteria. If so, the same was not included during data analysis. Moreover, IGRA is not a confirmatory test for TB as per WHO's recommendation and all IGRA positive patients should be subjected to other confirmatory tests.

We are very sorry for causing you such confusion. The main purpose of this study is to study the diagnostic efficacy of different pathogenic detection methods for tuberculosis. Others, such as IGRA, radiological results, and patients' clinical symptoms, only assist clinicians to make final diagnosis based on the results of etiology, so we do not make specific analysis, but choose to include them in the patients' baseline table (Page 10-11 Table 1) for a descriptive analysis. Please review again.

2.Role of IGRA and clinical features in screening the patients for TB in this study may be mentioned.

We are very sorry for causing you such confusion. The main purpose of this study is to study the diagnostic efficacy of different pathogenic detection methods for tuberculosis. Others, such as IGRA, radiological results, and patients' clinical symptoms, only assist clinicians to make final diagnosis based on the results of etiology, so we do not make specific analysis, but choose to include them in the patients' baseline table(Page 10-11 Table 1) for a descriptive analysis. Please review again.

3. Usefulness and practicality of this study in resource limited settings and low and middle income nations may be mentioned in terms of cost burden and how the early detection using tNGS could actually benefit the patients economically。

Thank you for raising the feasibility and practicality of tNGS in resource limited environments and low and middle income countries. In the discussion section, we have supplemented the cost-effectiveness of tNGS in MTB diagnosis and the specific

reasons for its application in low-income and high burden tuberculosis areas(Page 16 Line 29-Page 17 Line 14). Please double check.

4. Sociodemographic profiling of the study participants may be mentioned in a tabular format.

We are very sorry for causing you such confusion. The main purpose of this study is to study the diagnostic efficacy of different pathogenic detection methods for tuberculosis. Others, such as IGRA, radiological results, and patients' clinical symptoms, only assist clinicians to make final diagnosis based on the results of etiology, so we do not make specific analysis, but choose to include them in the patients' baseline table(Page 10-11 Table 1) for a descriptive analysis. Please review again.

5. Details about the resistance patterns and detection of loci for resistant genes may be mentioned if observed. It would be great if the same could be associated with socio-demographic factors

Thank you for your valuable insight on our manuscript. We have supplemented the consistency analysis of tNGS and Xpert on rifampicin resistance in the discussion section, and added the content of rifampicin resistant mutations discovered by tNGS, aiming to enrich the conclusions of the article(Page 20 Line 20- Page 21 Line 5). Thank you for checking again.

Reviewer 2：

1.The introduction would benefit from greater specificity and a clearer articulation of the study's objectives as they relate to the results presented.

Thank you for your insightful query demanding our correction of the study’s objectives. We have used more specific and clearer language to express the research’s objective(Page 3 Line 29-Page 4 Line 13). Thank you for checking again.

2.Although this is a retrospective analysis, have the authors done a post-hoc power calculation performed to validate the statistical significance of the study's sample size?

We appreciate you bringing your concerns to our attention. Nevertheless, we contend

that this research serves not only as a retrospective analysis but also as a diagnostic investigation. Therefore, efficacy analysis should not be employed to interpret historical clinical data. Furthermore, our objective is to accurately represent the diagnostic effectiveness of each detection method for a specific population over a defined timeframe. We acknowledge, however, that studies conducted at a single center may introduce selection bias and lead to certain distortions, which is recognized as one of the limitations of our study. Thank you for your understanding!

3.The authors need to specify the sequence read threshold parameters used in tNGS for determining MTB presence/absence, and clarify whether factors such as sequencing depth influenced diagnostic classification. Including a detailed tNGS result decision framework would enhance reproducibility and reader understanding.

Thank you for your insightful comments in our manuscript. We have addressed your concerns in the section of Material and methods(Page 8-9 2.7 Threshold parameters and decision framework for tNGS results). Plesase check again.

4.Consider including a subgroup analysis of MTB and NTM coinfection cases.

We appreciate your suggestions. However, according to our research design, patients with tuberculosis are classified in the positive category, whereas non-tuberculosis patients, including those with NTM infections, are categorized as negative. Our focus is solely on the differential diagnosis between these two categories using various testing methods. Including tuberculosis patients with NTM infections would hinder our ability to assess the diagnostic efficacy of each method. Consequently, we have decided to exclude these patients during the admission and exclusion phase. Thank you for your understanding.

5.The authors need clarify the reference standard used for sensitivity calculations. Were all diagnostic tests compared against culture as the gold standard?

We apologize for not specifying the benchmark utilized to assess the diagnostic performance of all detection methods, which impacted your review process. We have included the necessary explanations in the results section of the revised manuscript(Page 11 Line 4-7). Thank you for checking again.

6. It would be valuable to note any cases where tNGS failed to detect MTB that was identified by other diagnostic methods.

Thank you for your feedback to us. We have added this content to the discussion section(Page 18-Page 19). Thank you for checking again.

7. Beyond reporting positive detection rates, consider including sensitivity and specificity analyses for both sputum-positive and sputum-negative samples to demonstrate the added value of tNGS.

Thank you for your valuable feedback. Traditional pathogen testing negative pulmonary tuberculosis patients have always been the target of clinical doctors for early detection and treatment. We compared the positive detection rates of tNGS with other testing methods for smear-negative and culture-negative pulmonary tuberculosis patients through Tables 4 and 5(Page 14-Page 15), aiming to highlight the significant advantages of tNGS in this aspect. Thank you for checking again.

8. The discussion section would be strengthened by comparing your findings with other studies that have employed tNGS for MTB diagnosis and resistance analysis

Thank you for your insightful feedback on us. We have selected some representative references from recent years(such as reference 31 and 58) and compared them with our research results, aiming to improve the reliability of our research conclusions. Thank you for checking again.

9. Given the recommendation in the discussion section for use in low- and middle-income countries (LMICs), please address the cost-effectiveness of using tNGS solely for diagnosis when its primary advantage lies in resistance detection.

Thank you for raisingyour concern about the cost-effectiveness of using tNGS solely for diagnosis when its primary advantage lies in resistance detection. In the discussion section, we have supplemented the cost-effectiveness of tNGS in MTB diagnosis and the specific reasons for its application in low-income and high burden tuberculosis areas(Page 16 Line 29-Page 17 Line 14). Please double check.

10. While resistance analysis was noted as beyond the scope of this study,

including a concordance analysis of rifampicin resistance detection between Xpert and tNGS would add valuable insight and strengthen the discussion
Thank you for your valuable insight on our manuscript. We have supplemented the consistency analysis of tNGS and Xpert on rifampicin resistance in the discussion section, and added the content of rifampicin resistant mutations discovered by tNGS, aiming to enrich the conclusions of the article(Page 20 Line 20- Page 21 Line 5). Thank you for checking again.

11. The limitations section should be expanded to address how technical aspects of tNGS might impact its diagnostic accuracy.
Thank you for your important feedback. We have added some possible reasons for errors in tNGS in the limitations section of our discussion (Page 21 Line 10-17). Thank you for checking again.

12.Minor considerations - The authors can review the manuscript for the consistent use of past tense throughout, as this is a retrospective analysis.
Thank you for your careful consideration. We have reviewed the manuscript for the consistent use of past tense throughout. Please check again.
