# Supplementary material for: Nanopore-based targeted next-generation sequencing (tNGS): A versatile technology specialized in detecting low bacterial load clinical specimens
Source: PLoS One. 2025 May 27;20(5):e0324003. doi: 10.1371/journal.pone.0324003 (PMC12111578; doi:10.1371/journal.pone.0324003)
Supplement: Attachment — Submitted filename: response_to_reviewers_auresp_2.pdf [file pone.0324003.s004.pdf]

Dear Editor:

Thank you for your hard work in reviewing this research. We have uploaded the figure files involved in this study to the Preflight Analysis and Conversion Engine (PACE) digital diagnostic tool, account: 751289898@qq.com. Please check it.

Dear Reviewers:

The following are the responses to the reviewers. The changes in our revised manuscript are highlighted in blue, and the locations of corrections or supplements are specifically indicated in the corresponding responses. Thank you for your hard work in reviewing the manuscript.

1. Shifting the comparison of rifampicin resistance patterns between tNGS and Xpert from discussion to result section with a concise mention of these comparative insights in broader diagnostic context in the discussion.

Thank you for your insightful query. We have added 3.7 Evaluation of tNGS detection of rifampicin resistance patterns in the Results section of the article (Page 16 Line 5-Line 12). And the distribution of rifampicin-resistant mutations detected by tNGS on the rpoB gene was shown using a sunburst chart. In addition, in the discussion part of the article, we highlighted the advantages of tNGS in detecting rifampicin-resistant mutations outside RRDR compared with Xpert MTB/RIF, and the advantages of tNGS in providing comprehensive drug resistance information in the context of a wider range of drug-resistant tuberculosis (Page 20 Line 23-Page 21 Line 15). Please check again.

2. In discussion section, comparing the cost effectiveness to other WGS platforms or culture DST and mentioning how authors envision cost reduction when using tNGS in LMICs.

Thank you for your insightful advice. In the article's discussion section, we elaborated on the cost-effectiveness of tNGS compared to other detection methods for identifying mycobacteria and resistance mutations(Page 21 Line 16-Page 22 Line 6); additionally, we envision how to use tNGS to save costs in low- and middle-income countries(Page 22 Line 7-Line 19). Please check again.

3. Comprehensive grammar review

We have checked the article's grammar using Grammarly. Thank you for checking again.
